# The information and support needs of patients discharged after a short hospital stay for treatment of low-risk Community Acquired Pneumonia: implications for treatment without admission

**DOI:** 10.1186/1471-2466-8-11

**Published:** 2008-07-29

**Authors:** Deborah J Baldie, Vikki A Entwistle, Peter G Davey

**Affiliations:** 1 Social Dimensions of Health Institute and Alliance for Self Care Research, Universities of Dundee and St Andrews, Dundee, UK; 2 Health Informatics Centre, University of Dundee, Dundee, UK

## Abstract

**Background:**

There is increasing evidence that patients with low-risk community acquired pneumonia (CAP) can be effectively treated as outpatients. This study aimed to explore patients' experiences of having pneumonia and seeking health care; their perceptions of the information provided by health professionals; how they self managed at home; their information and support needs; and their beliefs and preferences regarding site of care.

**Methods:**

We conducted qualitative, semi-structured interviews with 15 patients who had a confirmed diagnosis of low-risk CAP and had received fewer than 3 days hospital care. Interviews were audio recorded and transcribed, and data were analysed thematically.

**Results:**

Most patients left hospital with no clear understanding of pneumonia, its treatment or follow-up and most identified additional-other specific information needs when they got home. Some were unable to independently address their activities of daily living in their first days at home.

Main concerns after discharge related to the cause and implications of pneumonia, symptom trajectory and prevention of transmission. Most sought advice from their GP in their first days at home, and indicated they would have appreciated a follow-up phone call or visit to discuss their concerns.

Patients' preferences for site of care varied and appeared to be influenced by beliefs about safety (fear of rapid deterioration at home or acquiring an infection in hospital), family burden, access to support, or confidence in home-care services. Those who received intravenous (IV) medication were more likely to state a preference for hospital care.

**Conclusion:**

Trends to support community-based treatment of CAP should be accompanied by increased attention to the information and support needs of patients who go home to self-manage. Although some information needs can be anticipated and addressed on diagnosis, specific needs often do not become apparent until patients return home, so some access to information and support in the community is likely to be necessary. Our finding that patients who received IV treatment for low-risk CAP were concerned about the relative safety of home-based care highlights the potential importance of the inferences patients make from treatment modalities, and also the need to ensure that patients' expectations and understandings are managed effectively.

## Background

About 1 per 1,000 of the UK adult population is admitted to hospital with community acquired pneumonia (CAP) each year at an estimated direct healthcare cost of £441 million [1].

There is increasing evidence that patients' risk of mortality from CAP can be quite reliably predicted using clinical severity assessment scores such as CURB65 [1-4], and many of those categorised as being at low-risk (CURB65 0 or 1) can be treated without admission to hospital with no adverse effects on outcomes [5-8].

Clinicians in the UK are increasingly encouraged to use clinical severity assessment tools and to treat low-risk patients with prescribed oral antibiotics as outpatients [1]. The acceptability of this strategy to patients is unclear, but patients in the US have been shown to be divided in their preferences for site of care and some have concerns relating to the safety of home care [9].

It is widely recognised that patients who are discharged from hospital with CAP have anxieties and information needs. Strategies such as the provision of standardised written and verbal information to patients prior to discharge from hospital have been shown to reduce anxiety levels and increase patients' knowledge of their condition and recovery [10,11]. Information provision alone, however, does not appear to eliminate patients' worries about the problems they may face once they are at home [10,11]. As we move towards community-based care for most low-risk CAP patients it is important that services learn how they can best support patients to self-manage this condition. Very little is currently known about: the concerns patients have when faced with the possibility of returning home from hospital assessment and/or treatment to self-manage; the challenges they face on returning home; and the information and support they do/would find helpful during their recovery period.

In Scotland a national improvement project was established to improve adherence to evidence based guidelines for managing patients with CAP, and to increase the number of patients with low-risk CAP who returned home to self-manage their recovery after hospital assessment. The project team sought to ensure that patients with CAP had optimal experiences of care and adequate information, advice and support during their period of self-management, but were unsure of the specific needs of patients.

This paper reports on an exploratory qualitative study that addressed the following:

• how do people with low-risk CAP experience hospital assessments and decisions about where and how they will be treated?

• how do they view and experience the possibility of going home to self-manage their CAP? and

• how do they view the information and support they were or might have been given to help them self-manage their CAP?

## Methods

Semi-structured interviews were used to collect information about the experiences of patients with low-risk CAP. We were keen to interview adult patients who had attended hospital and been diagnosed with low-risk CAP but also, according to best evidence, could have safely managed their recovery at home. We therefore sought to recruit adult patients (>16 years) who had presented at hospital, had a confirmed diagnosis of low-risk CAP (CURB 65 score of 0 or 1) and had no other co-morbidities that made hospital treatment necessary. The specific inclusion criteria included:

• written confirmation by a Specialist Registrar or Consultant in the medical notes of being treated for low-risk CAP (CURB65: 0 or 1) following a Chest X-ray showing infiltrates characteristic of CAP; and

• verbal/written confirmation by a Specialist Registrar or Consultant of no other co-morbidities requiring hospital care

Patients who were unable to take part in an interview due to dependence on oxygen, cognitive impairment or insufficient English were excluded from this small study. We aimed to interview those who were discharged from Accident and Emergency (A&E) or assessment units as well as those who had been admitted to hospital with low-risk CAP as we thought this would provide an insight into patient experiences of hospital assessment, immediate discharge to self-manage, information provision whilst in hospital and the types of concerns patients had once self-managing at home. Ethical approval was granted by the Tayside Committee on Medical Research Ethics 06/S1401/37.

### Sampling and recruitment

Purposive sampling was used to identify patients who had experience of attending/staying in hospital with low-risk CAP and returning home to self-manage their recovery. Patients were recruited from the medical assessment units of two teaching hospitals in the North East of Scotland between May 2006 and July 2007. In the larger assessment unit, DJB scrutinised the admission records on three days per week to identify patients who had been admitted in the previous 72 hours with any symptoms associated with CAP (e.g. cough, fever, acute confusion, breathlessness, chest tightness or productive sputum). She then screened all such patients' medical notes and confirmed their diagnosis and eligibility criteria with their Specialist Registrar or Consultant before approaching them to seek their participation in the study. This process was replicated by a specialist nurse three days per week in the smaller assessment unit.

Potential participants were given a verbal description of the study and an information leaflet. They were given at least 24 hours to consider taking part in an interview. People who had been sent home to self-manage before being approached by the researcher were sent a letter from their consultant which informed them of the study and invited them to contact the research team if they wished to take part.

### Data Collection

Face-to-face, semi-structured interviews were deemed appropriate to elicit the individual experiences of patients who had low-risk CAP as they would allow participants to talk about their experience in the context of their own lives [12]. Participants were informed of their right to discontinue their involvement at any time without their decision affecting any future care or treatment. Confidentiality was assured and written consent was obtained from each participant prior to each interview. Interviews were conducted 1–3 weeks following discharge, either in patients' homes or at a mutually convenient setting. An interview topic guide was used to ensure that all of the key research questions were addressed in each interview. This covered patients' accounts of: what happened prior to arriving at hospital, during assessment and hospital treatment, and after discharge from hospital; any information they were given during and after hospital treatment; their experiences of care (in hospital and/or at home); their involvement in decision making; how they coped at home following discharge from hospital; any concerns and information/support needs they had (in hospital and at home); their preferences regarding site of care (both at the time that it was decided and retrospectively/with the benefit of hindsight) and their recommendations about the kinds of information and other support that people with CAP need when they are discharged home. All interviews were audio-recorded and transcribed, and transcripts were checked for accuracy by DJB.

### Data Analysis

The Framework method [12] was used to order, synthesise and analyse the data. A number of key steps were followed to ensure a systematic analysis (Table 1).

**Table 1:** Framework Analysis Procedure (adapted from Ritchie and Lewis 2006[12])

1. **Familiarisation **is the process of becoming familiar with the range and diversity of the data. The research team read through the transcripts as they were completed, highlighting recurrent themes and generated a multitude of descriptive codes.

2. **Identifying a thematic framework**. Using the research notes from the first 6 transcripts the team identified key issues, concepts and themes with which to reference the data. An initial rough index was developed and applied to the transcripts. Further refinements were agreed by the research team as the index was applied to the transcripts. This continued up to an including transcript 9 with no further refinements required thereafter.

3. **Indexing**. This framework was systematically applied to the entire data set where each section of text in each transcript was labelled

4. **Charting **The researchers then attempted to build up a picture of the data as a whole, by rearranging summaries of material relating to each theme according to their thematic reference. Each participant had one row on the chart whilst themes were arranged in columns.

5. **Mapping and interpretation**. Once the data had been sifted and charted according to core themes, the researchers attempted to pull together key characteristics of the data, and to map and interpret the data set as a whole.

Two authors (DJB, VAE) read each transcript as interviews were completed and identified a list of key themes. The list was refined through repeated reading of transcripts and regular discussions about ideas or issues arising. This refinement continued until transcript 9 with no further adjustment required thereafter. The transcribed interviews were then systematically labelled using the thematic framework and summaries of material relating to each theme were then entered onto thematic charts with rows for each of the participants. The authors then used the thematic charts to develop descriptive summaries and look for key patterns in the data.

## Results

All patients identified as eligible to take part were followed up and either approached by the researcher or, in the cases where they had already been discharged, sent a letter inviting them to take part in the study from their consultant. We were unable to recruit any patients who had presented at A&E and went home to self-manage. 59 patients (21 female, median age 69) were identified as eligible to take part. No-one was excluded on grounds of insufficient English. 54 were invited to take part by DJB or the specialist nurse and 5 were sent an invitation by their consultant. 20 of those invited by the research team subsequently returned a form initially indicating their willingness to participate and 15 were interviewed.

Because of our approach to recruitment and the limitations of hospital information systems we were unable to accurately detect all patients with low-risk CAP presenting to the service during the data collection period and therefore cannot indicate how representative our sample is of the patient population.

The 15 people who participated included 8 women and 7 men aged between 22–74 years (median age 59). All had experienced a short stay in hospital (2–3 days) for treatment of low-risk CAP before returning home, and 5 received intravenous antibiotics in the first 24–48 hours. Eight participants reported that they had pre-existing long term conditions (asthma (3), COPD (3), heart condition (1), osteoporosis (1)). Most (11) lived with a partner/spouse, 2 lived with parent(s), one was a single parent and one lived alone.

### Key findings

Few patients who attended hospital for assessment and treatment for low-risk CAP recalled having a say about where they were treated. Most recalled having received some information about their condition and its treatment while they were in hospital. Most also identified a number of information and support needs. These were often personally specific, and some were identified only after they returned home. Once home, despite having been in hospital for 2–3 days, many found it difficult to self-care in their first few days and needed help from family with dressing and household chores.

Participants tended to be equally divided in their preferences for site of care and cited issues such as safety, burden on family and comfort when considering their preference. Some participants indicated that self-care at home would be viewed as more attractive if they were given clear explanations of their illness and informed on how to manage symptoms. They also suggested that they would have felt more reassured if a health professional had checked on their recovery and if they had access to someone who could answer any questions they had.

### Patients' experiences of referral to hospital and hospital assessments

All participants were registered with a general practitioner (GP), which is typical for Scotland. When they were troubled by the symptoms of what was subsequently diagnosed as low-risk CAP they initially sought support from their GP, NHS 24, a national out of hours health advice service , local out-of-hours services or A&E. Regardless of where they first presented for health care support participants perceived that they had been referred to hospital with no/little indication of the likely diagnosis or what might result from that hospital visit. They perceived little involvement in the decision to admit them with only one recalling being given the option to return home. Some indicated, however, that this was not an issue for them: they had expected to be admitted because they had been referred to hospital. They also often felt too ill to listen to information provided and were content for hospital doctors to make that decision on their behalf.

### Information provided during hospital stay

Information provided during hospital stay tended to be rated poorly. Over half of the participants told us they left hospital with no clear understanding of pneumonia, and some talked of limited opportunities to discuss their condition, its treatment and their concerns. No-one recalled being given a written patient information leaflet (although the services did have these).

Participants often perceived nursing and medical staff as too busy to explain their condition to them, and some highlighted that ward rounds were not conducive to them being involved in discussions that concerned their treatment/recovery.

*"More the doctor talked to whoever was with them, whether it was the doctors or the nurses and not actually talked to yourself, ... they would go talk to, whether it was medical students or whoever it was they had with them, it's so and so and so. And we've got to do this and that and that. So you're just basically listening to what they're saying to them, not what he's saying to me, sort of thing." *(Int 14, 587)

A few participants recognised that some of the information they needed probably had been provided at assessment or shortly after admission, but they had either felt too ill to listen at the time or had since forgotten.

*"There was a lot of discussions going on, my wife was there the whole time, and there was doctors out and in, there was nurses there, I don't remember huge amounts about what was said, I didn't pick up an awful lot, I was just feeling so tired and confused at the time and I picked up right away, yes, pneumonia, right along and that's...They were speaking to me but she (wife)was there, sort of taking it all in more than me, I think she sort of, whether they realised and she realised I was not taking it all in." *(Int 5, 44).

A small number (4/15) of participants thought that they had received sufficient information and advice prior to discharge. Three indicated that they had been provided with little information but were content with that as they had either previous experience of the illness, had no perceived information needs or felt they could ask their GP once home. One however indicated that the information they received was excellent.

*"... I knew everything that I got to do, I'd got to take it easy, I've not got to do anything stupid like get myself exhausted again, and I knew precisely what I'd got to take in terms of the doses of the drugs... so I knew exactly, I mean, three people had been through it with me, what I had to take..."*(Int 3, 392)

These individuals felt fully equipped to return home and self-manage.

### Self-managing at home

Most participants told us that the main symptom they had to manage was extreme tiredness, often with sudden onset.

*"..I could be bright as Larry, I could be sitting, as I say, watch something and the next thing I'm going (to sleep)... that's all I'm doing. As I say, all I'm doing is resting" *(Int 2, 12)

*"The first couple of days (at home) I thought I was alright, but I'm not alright....one day, I got up, had a shower, and I mean that was fine, I had a lot of paperwork to do, but no, I didn't, I fell suddenly asleep again" *(Int 3, 247)

Despite having all spent a short time in hospital; many needed considerable support once home. Some thought that they would not have been able to manage at home without the support of their spouse/partner as they needed help to wash, dress, and cook and clean. For example:

*"I have my husband at home, but if I had been coming home and had nobody here, I couldn't have coped, ...... I couldn'ae pull my jumper off, I was just so sore, awful tired... the first week or so I wouldn't have felt like cooking or doing nothing, ... I didn't think you could cope on your own when you first come home..." *(Int 8, 376)

### Information and support needs of patients once home

**Figure 1:**
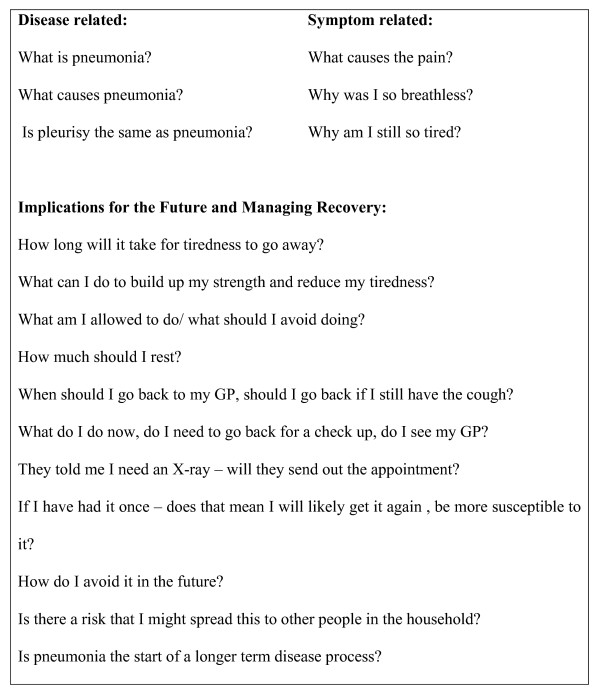
Information needs reported by patients who have returned home.

Whilst some patients recalled that they had wanted more information prior to their discharge, most indicated that it was not until they returned home that they had any questions or concerns. They told us that since returning home they had realised that they had little understanding of the origins, meaning and implications (short and long-term) of pneumonia, or what constituted a normal recovery. They had no clear understanding of medical follow-up; how to best manage symptoms; how to recognise deterioration or lack of response to treatment; or how to avoid catching or spreading infection from/to others. Participants' concerns are listed in Figure 1.

A significant proportion (10/15) contacted their GP in their first 2–3 days after discharge to seek advice on these issues. Those who did not contact their GP had either previously experienced pneumonia or had a chronic disease and felt able to self-manage. Some participants also sought or were given advice by friends and family. Participants who had received information from friends tended, however, to have inaccurate expectations of how long it would take them to recover, believing it might take them months or up to a year to fully recover.

### Preferences for site of care

All participants were asked to consider whether and why they would prefer hospital or home when given a diagnosis of CAP that required oral antibiotics and rest. Participants were reasonably equally divided in their preference for home or hospital as site of care. Eight indicated that with hindsight they would have preferred home, 5 would have preferred hospital and 2 were undecided. Of the 8 who had a stated preference for home, 6 indicated they would have gone home if given that choice during their assessment. The two others indicated that with hindsight, they would have preferred to return home to self-manage, but that at the time of admission they had felt very ill and had been happy to be admitted.

All of those who stated a preference for hospital had concerns that they might deteriorate rapidly and so preferred to be in hospital where their condition could be professionally monitored and they could readily access emergency help.

*"I wouldn't have felt comfortable with that [being treated at home], maybe because I had the extra bit with oxygen but I felt happier where I was, with the risk of anything happening." *(Int 11, 470)

*"..it was just nice to know that if anything had gone wrong, I was in a hospital. I don't know if everybody feels the same when they've got pneumonia, but I was definitely in a lot of pain, so I would have rather been in the hospital for them to be able to check on me. Because at home, I just, I think I would probably worry even more if I was at home with it*." (Int 11, 629)

Participants did not routinely refer to their experiences of treatment as a reason for their preferences but all of the 5 patients who had received IV antibiotics and oxygen in their first 24 hours of hospital stated a preference for hospital care. It is possible that treatment type may have influenced patients' perceptions of how ill they were and subsequently their beliefs about the most appropriate site of care.

"..*depends if you're needing oxygen, are they going to give you oxygen at home? *" (Int 7, 1144)

*"if you were at home, you wouldn't get the various drugs as quickly... whereas in a hospital you've got everything, haven't you? It doesn't matter what happens. They've got it on hand..." *(Int 3, 424)

Conversely, those patients that received oral antibiotics saw no added benefit to hospital and stated a clear preference for home.

*".. well, what I was doing in hospital I could do at home, I mean I was only resting there and taking my pills..." *(Int 8, 630)

Those patients that had previously self-managed acute exacerbations of an existing lung condition at home had a better understanding of the treatments and support they could access at home and appeared more confident to manage treatments such as nebulisers at home.

*"Well, ..on the Sunday night, the doctor... wanted to admit me ....but ... I'd prefer to come home and stay here rather than go to hospital *(Int 10, 172)...*you know yourself when you need to take it [nebuliser].... just even knowing that the nebuliser is here is just reassuring so that helps you as well.. *(Int 10, 421)* ... I've got my asthma nurse during the day, Monday to Friday, at any other time, out with that I've got NHS 24 and I wouldn't think twice about phoning them." *(Int 10, 609)

The level of social support participants perceived they had access to also appeared to influence participants' preferences for site of care. Some participants with dependants at home preferred to stay in hospital and avoid burdening them or other family members.

*"At my age, stage of life, and the circumstances of my mother being at her age, I'd rather be in hospital for it, because then I know it would be a few days in there and my mum wouldn't have to worry about looking after me... Because at her age, it's not fair to put any burden like that on a person at that age." *(Int 6, 22)

Participants who stated a preference for home offered a variety of reasons for this preference. Some perceived that hospital provided nothing more than home. The reason for preferring home provided by most participants however was the comfort and privacy that it provided.

*"I think you just feel more comfortable at home and you're more at ease at home and you can, you know, please yourself, even like go for a bath to help, and just get up and wander about and you know, please yourself if you're at home..."*(Int 10, 246)

Those who had been happy to have been admitted when they felt ill but with hindsight preferred home said that they had had little opportunity to rest in hospital and now believed that they would have recovered quicker at home.

Just as some patients perceived hospital care to be safer than home care, others viewed home as the safer site. Several mentioned a concern to avoid hospital acquired infections.

*"I didn't want to catch an infection, you go where they breed infection and that's the place they'll breed them." *(Int 15, 398)

When asked what would make them more confident to self-manage at home, most suggested some form of monitoring by a health care professional. They saw this as a way of increasing peoples' understanding of how well they were progressing. Some also suggested that having someone they could contact by phone to ask questions related to their recovery/response to treatment would provide reassurance.

*" ..if you're at home, you're always thinking, oh is this right, am I getting worse, am I getting better, oh, I wish I could speak to somebody and ask them a question, ...at home, ... you're always phoning the GP, you think, oh, he's bothering me again, but, am I feeling, is this right, is this wrong, no, oh no, you're okay, that's okay, you think, oh, sorry to bother you again." *(Int 5, 718)

## Discussion

Our study is the first to report the self-perceived information and support needs of patients discharged from hospital following treatment of low-risk CAP. It has shown that most of these patients do have significant information needs once they return home to self-manage their recovery. Some also find it difficult to self-care in their first few days and may need significant support from family members. People vary in their preferences for site of care, and their preferences are influenced by their beliefs about safety, concerns about the burden on their family, access to family support and awareness of (and confidence in) home-based care services. Some participants suggested that a review of their progress and access to a telephone advice service would make self-care a more attractive option.

### Strengths and limitations

This was a small study of 15 patients which excluded carer's perspectives and patients who could not speak English. Nevertheless the sample included men and women of various ages, with and without a previous long-term condition. Difficulties with recruitment prevented us from being able to recruit patients who went home to self-manage without an admission to hospital, but the study provides important insights into how patients cope with recovery from low-risk CAP at home after only a few days in hospital and how perhaps they might be better advised and supported during their period of ill health. Participants were interviewed within 1–3 weeks after discharge from hospital, so potential problems associated with the recall of events were minimised. It seems likely that people who are and are not hospitalised will have some similar information needs and anxieties about managing their CAP at home, but further research is needed to examine the experience of patients who self-manage without any hospitalisation.

It is possible that our interviews tended to over-identify information needs because participants may not have had concerns about the information they were given until we asked them to consider this in an interview. However, the fact that several had sought professional advice or health care support relating to their concerns prior to their interview suggests that this was not the case.

### Information needs of patients

Most participants had significant information needs but did not realise this until they returned home. Their information needs mainly related to their clinical condition, treatment, implications and medical follow-up. Regardless of medical condition, these types of information needs have previously been found to be rated highly by patients in terms of their importance before and after discharge and become increasingly important to patients in the few weeks after discharge [13].

We also found that information needs differed between participants and many contacted their GP to seek reassurance and advice within the first 2–3 days of being at home. Their lack of knowledge about their condition, follow-up and recovery may be related to the quality and volume of information they received in hospital, but information needs can also arise at home. Patients are sometimes unsure of what to ask when in hospital, [14] their ability to understand information differs in accordance with the stage of their illness, and they may find it difficult to recall information they are provided with during their hospital stay [15-17].

It is clear that as increased numbers of patients with low-risk CAP are treated at home efforts must be made to ensure they have adequate information and support. It may help to give patients information in both written and oral form and repeat it as required within hospital. However, having access to personal advice once home appears critical. CAP information leaflets do not fully address patients' information needs [11] and patients find it difficult to use the standard information they receive and value being able to access someone who can answer their queries [18]. We found this to be the case amongst patients we interviewed with some suggesting that they feel they would have benefited from being able to call someone with professional expertise.

Patients' primary care teams are well placed to help them identify and address their concerns and uncertainties and thus assist them to feel more empowered to self-manage. Prompt communication between hospital and community staff is required, however, if such patients are to be made known to, and given appropriate support by their primary care team.

### Self-managing at home

Empirically it is accepted that for most patients with low-risk CAP, home care is as effective as hospital care. Until now however we have had little understanding of how patients cope at home. We have highlighted that many patients were surprised at the level of tiredness they experienced and the extent to which they had to rely on others in their first few days for help with dressing, personal hygiene and domestic chores.

It is recognised that older patients require support with their activities of daily living and housework in their first week home from hospital [19]. We found that patients of all ages needed support which most received from their family. Any extension of home care for patients with low-risk CAP will therefore require hospital practitioners and others who diagnose low-risk CAP to rapidly assess the support needs of patients and help to secure immediate, short-term assistance with their basic activities of daily living if necessary. Research into the impact and needs for family whilst caring for patients at home may also help shape future care models for this group of patients.

### Preferences for site of care

People arrived at hospital ill-prepared about what to expect and tended to be unaware of the option to return home and self-manage. Our study suggests that patients who experience IV antibiotics have anxieties about home as a safe site of care and tend to prefer hospital as a site of care. A previous study has reported that patients with low-risk CAP who had experienced IV treatment within an extended care at home scheme were happier with their location of care than those who received hospital care [20]. It is possible that preferences in our study, as in many other studies, were influenced by the patients' experiences of health care, their beliefs about what is possible and safe and/or their health care expectations. Further exploration into the experiences and preferences of patients who go home from assessment units to self-manage (without admission to hospital), with no extended care at home scheme may help practitioners better understand and address their concerns.

## Conclusion

Patients who visit hospital with low-risk CAP return home with considerable information needs and many need physical support with activities of daily living and housework in their first few days home. They are often uncertain if their recovery is in line with what is expected and may be particularly concerned about how they can best support their recovery and avoid future infections. Some want information on the short and long-term implications of their disease.

Practitioners (GPs, hospital doctors and nurses) might better support these individuals by providing clear information repeatedly throughout the care journey. Information given after hospital discharge may ensure patients receive information at a time when they are more aware of their own needs and best able to receive information and advice.

Access to and initiation of immediate short-term support for activities of daily living is necessary if practitioners are to ensure older patients or those with dependants are well supported to safely self-manage their recovery at home.

## Competing interests

The authors declare that they have no competing interests.

## Authors' contributions

DJB carried out all the interviews and data analysis. VAE supervised the project, read all transcripts and supported data analysis and helped draft the manuscript. PGD was a clinical collaborator and approved the final manuscript.

## Pre-publication history

The pre-publication history for this paper can be accessed here:


